# Pulmonary Langerhans Cell Histiocytosis Masquerading as Lymphangioleiomyomatosis

**DOI:** 10.7759/cureus.38486

**Published:** 2023-05-03

**Authors:** Priya Patel, FNU Anamika, Rana Ali

**Affiliations:** 1 Sleep Medicine, Hackensack Meridian Health Ocean University Medical Center, Brick Township, USA; 2 Internal Medicine, Hackensack Meridian Health Ocean University Medical Center, Brick Township, USA; 3 Pulmonary and Sleep Medicine, Hackensack Meridian Health Ocean University Medical Center, Brick Township, USA

**Keywords:** lam, plch, diffuse lung disease, cigarette smoking, lung cysts

## Abstract

Pulmonary Langerhans cell histiocytosis (PLCH) is an uncommon lung disease that affects young adults aged 20 to 40 years with current or prior history of smoking. The pathologic cell type in PLCH is a dendritic cell of the monocyte-macrophage line that resembles cutaneous Langerhans cells. This report presents the case of a 42-year-old woman with PLCH. We discuss her clinical symptoms, diagnostic tests, and treatment plan, with a specific focus on the radiologic features. The patient exhibited a radiologic appearance similar to that of lymphangiomyomatosis with histologic evidence of PLCH.

## Introduction

Pulmonary Langerhans cell histiocytosis (PLCH) is an uncommon cause of diffuse parenchymal lung disease that represents 3% of all causes of interstitial lung disease [1]. It is a rare histiocytic disorder that almost exclusively affects the lungs of smokers. It is thought to be an inflammatory myeloid neoplasm. The pathologic cell type in PLCH is a dendritic cell of the monocyte-macrophage line that resembles cutaneous Langerhans cells [2]. This case report provides a comprehensive review of the clinical, radiologic, and diagnostic features of PLCH in a 42-year-old female patient.

## Case presentation

**Figure 1:**
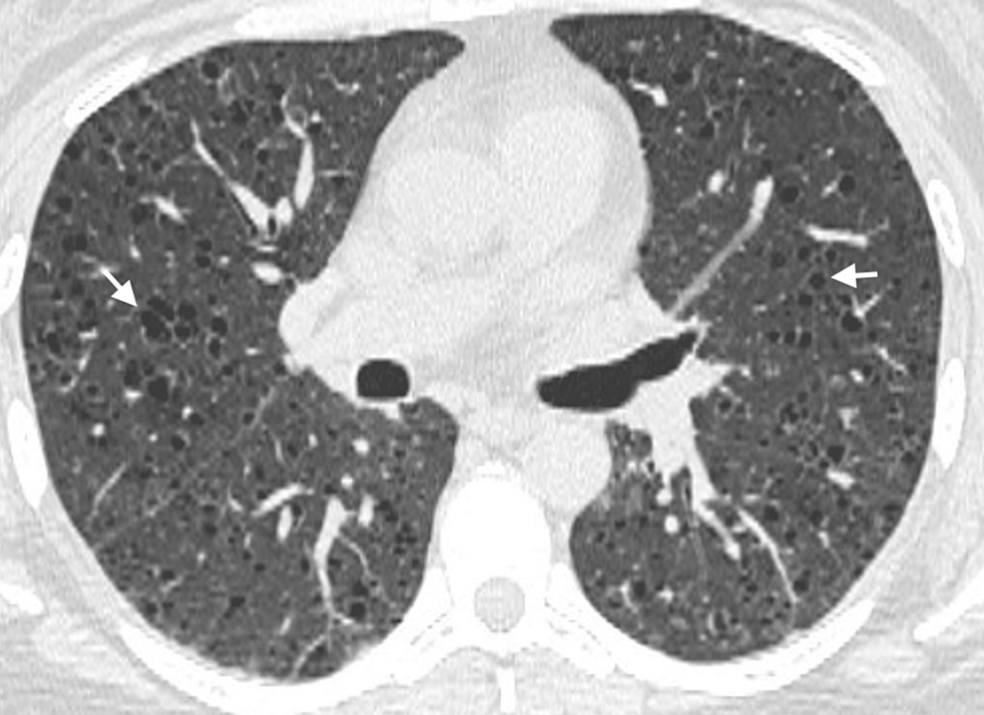
A high-resolution computed tomography of the chest of the patient displayed in the lung window showing small round bilateral cysts (white arrow).

A 42-year-old female with a 15-pack-year history of smoking sought medical attention at the clinic because a computed tomography (CT) scan of her abdomen, which was done to investigate her renal colic, revealed abnormal lung findings. It showed cystic changes at the base of her lungs, with no evidence of renal angiomyolipomas. She had symptoms of mild dyspnea on exertion and weight loss, with a normal physical examination. She had no other significant past medical history. Her family history was notable for lung cancer in her father and gastric cancer in her brother. Her only medication was birth control which she was on for 21 years. A pulmonary function test (PFT) showed normal lung functions with a normal diffusing capacity of the lungs for carbon monoxide (DLCO). A high-resolution computed tomography (HRCT) of the chest showed innumerable small round cysts bilaterally and evenly distributed from apex to base, as well as small pulmonary nodules and lymph nodes within the mediastinum (Figure 1).

These distinctive radiographic features were most suggestive of lymphangiomyomatosis (LAM), and a provisional diagnosis of LAM was made. However, PLCH was also in the differential for similar cystic lung changes and the presence of pulmonary nodules with her extensive smoking history. Further workup with serum vascular endothelial growth factor-D (VEGF-D) level was considered for LAM; however, due to limitations imposed by insurance, a definitive diagnosis could not be obtained at the time. Given her normal lung functions, she was managed with complete abstinence from smoking and avoidance of exogenous estrogen for PLCH and LAM, respectively. Unfortunately, she was then lost to follow-up.

She returned to the clinic as her respiratory status had declined over the past five years with worsening dyspnea on exertion, persistent cough, and recurrent episodes of bronchitis requiring antibiotics. She had been on steroids and long-acting bronchodilator inhalers for her current symptoms. She was also placed on oxygen for nocturnal hypoxia. PFT was performed which showed mild obstructive lung disease with forced expiratory volume in one second (FEV1) of 64% and reduced DLCO of 52%. On the six-minute walk test, she was able to maintain her oxygen saturation above 97% but had tachycardia and shortness of breath. A repeat CT of the chest showed no significant interval change in the extensive cystic lung disease and pulmonary nodules compared to her initial imaging. Due to the progression of her symptoms, video-assisted thoracoscopic surgery with left upper lobe wedge resection was performed to obtain a definitive diagnosis and formulate an appropriate treatment plan. The histological analysis showed emphysematous changes and respiratory bronchiolitis, cystic areas associated with fibrosis and smooth muscle in the wall, and stellate scar-like areas, a few of which showed increased eosinophils along with lymphocytes, plasma cells, and histiocytes. On immunostains for S100 and CD1a, numerous Langerhans cells were present. Mutation for the *BRAF *gene was negative. Smooth muscle fibers were also negative for HMB45. Diagnosis of PLCH was established. A skeletal survey was done to evaluate extrapulmonary disease and revealed no lytic or blastic lesions. Her echocardiography showed no signs of pulmonary hypertension. She was treated with corticosteroids and six cycles of cladribine. She reported interval improvement in her symptoms of cough and dyspnea. A follow-up CT of the chest after treatment revealed a decrease in the size of bilateral pulmonary nodules with no significant change in the size of the cysts. Her PFTs showed a gradual increase in DLCO from 52% at the nadir of the disease to 71% post-treatment.

A few years following her treatment for PLCH, she developed a rash on her bilateral lower extremities which was non-responsive to oral and systemic corticosteroids. Subsequent skin biopsy was suggestive of extrapulmonary manifestation of PLCH and the patient was treated with vinblastine and 6-mercaptopurine over a course of four cycles. A positron emission tomography-computed tomography scan did not reveal any fluorodeoxyglucose-avid lesions. She was followed up in the office and had functional and symptomatic improvement in her overall status.

## Discussion

PLCH is most frequently an isolated disease process occurring in adult smokers between the ages of 20 to 40 years. The most common extrapulmonary features in PLCH patients are cystic bone lesions, diabetes insipidus, and skin lesions [3,4]. Initially, patients may present with nonproductive cough and dyspnea; however, some patients may be asymptomatic with only an abnormal chest radiograph. Another frequent way the condition manifests is through spontaneous pneumothorax, which tends to recur and may be bilateral [5,6]. The usual abnormality noted during the diagnosis of PLCH is a reduction in DLCO. PFT may also show obstructive, restrictive, or mixed physiological anomalies [1].

HRCT may be diagnostic in PLCH, particularly when a combination of cysts and nodules in a characteristic upper lung zone distribution is observed [7]. HRCT findings may vary based on the natural course of the disease. In the earlier stages, nodules (measuring from a few millimeters up to 2 cm) are common and may show central cavitation, whereas cysts tend to predominate in more advanced stages. The radiologic appearance of PLCH is characterized by the combination of nodular and cystic change, with cysts often irregular and sparing the lung base [1]. In contrast, LAM cysts have relatively uniform size and thin walls, are distributed evenly in a diffuse manner, and are separated by normal lung parenchyma [8]. The radiologic findings of our patient showed evenly distributed small round cysts with thin walls and mediastinal lymph nodes, leading to a high suspicion for LAM.

Further evaluation and definitive diagnosis should be pursued when treatment is contemplated. A transbronchial biopsy has been considered to have a low yield in the diagnosis of PLCH [9]. A lung biopsy can generally be avoided when HRCT findings are characteristic and concordant with the clinical history. However, in situations where the diagnosis is unclear, such as in this case, a surgical lung biopsy is necessary [10]. Transbronchial lung cryobiopsy is a newer technique for sampling lung tissue for interstitial lung disease diagnosis and is a promising strategy that may become an alternative to surgical lung biopsy; however, large-scale studies are required to investigate its reliability and diagnostic power compared to surgical lung biopsies [11,12]. On histological examination, early lesions are centered on terminal and respiratory bronchioles. In the early stages, Langerhans cell histiocytosis (LCH) cells, which stain for S100 and CD1a, can be seen infiltrating bronchiolar walls and epithelium. Later, the LCH cells are surrounded by variable numbers of lymphocytes, eosinophils, fibroblasts, and plasma cells progressing to characteristic stellate fibrotic scars enclosed by cystic areas [13,14]. Serum VEGF-D is a highly specific diagnostic biomarker for LAM [8], which was initially considered in this patient when the provisional diagnosis of LAM was made. However, it was not obtained due to fewer testing facilities and insurance limitations. The histologic analysis of the patient in this case report was consistent with numerous Langerhans cells on immunostains for S100 and CD1a and negative for HMB45, which is another immunohistochemical marker for LAM, thus confirming the diagnosis of PLCH.

The management of PLCH involves abstinence from smoking, which leads to symptom stabilization in many patients [1]. However, despite smoking cessation, the disease may progress, as in the case of our patient. Experts suggest that in the absence of evidence of a benefit, corticosteroid therapy should be reserved for symptomatic patients with predominantly nodular lesions on HRCT [5,15]. Chemotherapy agents have been reported in inducing disease remission, particularly in patients with isolated progressive pulmonary disease. Cladribine, a purine nucleoside, has been effective in both multisystem and isolated PLCH, but there is no evidence to support the reduction in the size of lung cysts in response to cladribine. For patients with *BRAF^V600E^* mutation, targeted inhibition of the BRAF/MAPK/MEK pathways, such as vemurafenib and trametinib, may be a viable treatment option [1]. However, our patient did not have the *BRAF *mutation and was treated with six cycles of cladribine, resulting in clinical improvement of her symptoms with no change in the size of the cysts.

The overall prognosis for PLCH is generally good, particularly if patients stop smoking early in the course of the disease. Poor prognostic factors include multisystem involvement, extensive cysts, markedly reduced DLCO, low FEV1/forced vital capacity ratio, and a high ratio of residual volumes to total lung capacity [1].

## Conclusions

This case report emphasizes the LAM-like radiologic appearance of PLCH, characterized by multiple small round cysts of relatively uniform size, shape, and distribution, in contrast to pleomorphic cysts that often spare the lung base seen in PLCH. While clinical features and imaging findings can aid in making a provisional diagnosis, biopsy tissue analysis may be necessary for an accurate diagnosis and treatment plan.
